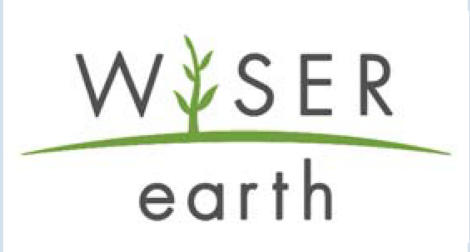# EHPnet: WiserEarth

**Published:** 2007-09

**Authors:** Erin E. Dooley

In 2004 the Sausalito, California–based Natural Capital Institute began developing the World Index for Social and Environmental Responsibility (WISER) to bring together the more than 1 million organizations working to improve environmental and human health. WISER has launched a website targeted specifically at nonprofits at **http://www.wiserearth.org/**. Business and government sites are planned for later.

WiserEarth provides tools and a platform for NGOs, funding sources, and individuals to network and develop awareness of each other’s work. Individuals can post personal profiles, and NGOs are given space to build a web presence.

Visitors can post events and job openings, or participate in discussion forums on such topics as holistic health, sustainable agriculture, and climate change. The website features 44 main areas of focus, termed “portals,” which are further subdivided into 372 more specific topics. Among the portals are agriculture and farming, greening of industry, fisheries, pollution, poverty eradication, and sustainable development. Each portal page links to organizations, users, resources, jobs, and events associated with that topic. The page devoted to organizations allows visitors to browse for information a number of ways including by country, type of organization (e.g., community-based organization, NGO), area of focus, and activity type (e.g., research, activism).

A news section features rundowns of the latest updates to the website, postings by the media and press center, new multimedia offerings, volunteer openings, and a calendar of upcoming events around the world. Each day the site spotlights a different portal, organization, resource, and event.

## Figures and Tables

**Figure:**